# Planned surgery in the COVID-19 pandemic: a prospective cohort study from Nottingham

**DOI:** 10.1007/s00423-021-02207-8

**Published:** 2021-06-15

**Authors:** J Catton, A Banerjea, S Gregory, C Hall, CJ Crooks, CA Lewis-Lloyd, A Marshall, DJ Humes

**Affiliations:** 1 Division of Surgery, Nottingham University Hospitals NHS Trust, Nottingham, NG7 2UH UK; 2 National Institute for Health Research (NIHR) Nottingham Biomedical Research Centre (BRC), Nottingham University Hospitals NHS Trust and the University of Nottingham, School of Medicine, E Floor West Block, Queen’s Medical Centre Campus, Nottingham, NG7 2UH UK

**Keywords:** Upper GI, Lower GI, HPB, COVID-19, Mortality

## Abstract

**Purpose:**

Globally planned surgical procedures have been deferred during the current COVID-19 pandemic. The study aimed to report the outcomes of planned urgent and cancer cases during the current pandemic using a multi-disciplinary prioritisation group.

**Methods:**

A prospective cohort study of patients having urgent or cancer surgery at a NHS Trust from 1st March to 30th April 2020 who had been prioritised by a multi-disciplinary COVID Surgery group. Rates of post-operative PCR positive and suspected COVID-19 infections within 30 days, 30-day mortality and any death related to COVID-19 are reported.

**Results:**

Overall 597 patients underwent surgery with a median age of 65 years (interquartile range (IQR) 54–74 years). Of these, 86.1% (514/597) had a current cancer diagnosis. During the period, 60.8% (363/597) of patients had surgery at the NHS Trust whilst 39.2% (234/597) had surgery at Independent Sector hospitals. The incidence of COVID-19 in the East Midlands was 193.7 per 100,000 population during the study period. In the 30 days following surgery, 1.3% (8/597) of patients tested positive for COVID-19 with all cases at the NHS site. Overall 30-day mortality was 0.7% (4/597). Following a PCR positive COVID-19 diagnosis, mortality was 25.0% (2/8). Including both PCR positive and suspected cases, 3.0% (18/597) developed COVID-19 infection with 1.3% at the independent site compared to 4.1% at the NHS Trust (*p*=0.047).

**Conclusions:**

Rates of COVID-19 infection in the post-operative period were low especially in the Independent Sector site. Mortality following a post-operative diagnosis of COVID-19 was high.

**Supplementary Information:**

The online version contains supplementary material available at 10.1007/s00423-021-02207-8.

## Introduction

It is estimated over 28 million operations will be deferred globally during the COVID-19 pandemic peak [1]. Our understanding of the consequences of developing acute respiratory syndrome coronavirus 2 (SARS-CoV-2) infection in the post-operative period is not yet clear, with initial reports suggesting mortality in excess of 20% in confirmed cases [2]. International and National guidance on the management of patients in the perioperative period has changed during the course of the pandemic in response to emerging evidence, but it remains extremely limited in many areas of clinical concern [3–5]. Non-urgent surgical cases were suspended for 3 months in the UK in April 2020 due to concern that NHS capacity might be overwhelmed by patients requiring respiratory support [6]. Patients requiring urgent and cancer care were advised surgery would be undertaken but might be delayed depending on the local infection rate and COVID-19 burden on services. This was set against the potential risk of harm due to delays in cancer surgery [7].

In response to this developing crisis, we formed a multi-disciplinary COVID Cancer Surgery group at Nottingham University Hospitals NHS Trust (NUH) to oversee business continuity plans and prioritisation of these clinically urgent and cancer cases. This group not only addressed prioritisation of cases but also the allocation of constrained resources such as anaesthetic staffing, post-operative critical care capacity, availability of theatre and recovery staff and theatre space across two NHS hospital sites and two Independent Sector hospitals in Nottinghamshire. Additional factors for consideration that emerged included availability of appropriate levels of Personal Protective Equipment (PPE), safety in relation to aerosol generating procedures (AGPs), access to COVID testing as well as anaesthetic drugs and the need for higher levels of out of hours medical care in remote sites. Ethical considerations were addressed with input from members of the Trust’s Ethics Committee with inclusion of lay representatives at every meeting.

We aim to report the process for forming this committee alongside the clinical outcomes of planned surgery in Nottingham during the peak of the pandemic.

## Material and methods

### Setting

Nottingham University Hospitals (NUH) NHS Trust is a large teaching hospital which normally serves as a tertiary referral centre and trauma centre. It is set across 2 campuses with 42 operating theatres and 1300 beds. BMI The Park Hospital and Spire Nottingham Hospital are Independent Sector sites.

### COVID Cancer group

The group was convened by the Deputy Medical Director, chaired by the Lead Cancer Clinician, and initially consisted of lead cancer nurses, corporate operations representatives, senior anaesthetists, critical care consultants and clinical representatives of Divisions that undertake cancer surgery in our Trust. All cancer related and perioperative pathway specialties were co-opted into the prioritisation group including breast, colorectal, hepatobiliary, endocrine, gynaecology, head and neck, neurosurgery, plastics, thoracic, upper gastrointestinal surgery and urology, with additional support from microbiology/infectious diseases consultants, administrators and ethics representatives for the Trust. Each service had already been asked to identify all patients known to be awaiting cancer surgery with a documented cancer Multi-disciplinary team (MDT) outcome and to prioritise them according to clinical need and the availability or otherwise of reasonable alternatives to elective surgery. This was soon expanded to clinically urgent cases, including operations or procedures that might prevent emergency admission as per National guidance. New cancer (and clinically urgent diagnoses) was prospectively added after appropriate MDT review.

All prioritised cases were submitted to the group and logged prospectively. All outcomes were audited to ensure that there was no excess COVID-related morbidity and mortality and that outcomes in the Independent Sector were comparable to the NHS Trust. The Group passed through a number of phases in response to the pandemic curve and alterations in National and Local guidance. All patients were consented for their appropriate procedure in line with Trust standard operating procedure but with the additional risk of infection with COVID-19 and its associated complications including an increased risk of mortality.

The group formed a framework for this decision-making process (Supplementary Figure 1 and Online Resource 1). Decisions were based around the possibility of delay, or other treatments, the proposed benefit of surgery with likelihood of cure, the complexity of the surgery and individualised patient factors. These decisions were then set in the context of local COVID-19 infection rates, theatre capacity, critical care capacity and efficiency considerations.

### Phase 1

The first documented inpatient case of SARS-CoV-2 at NUH was in late February 2020. In the initial phase of the pandemic when case numbers were low planned surgery continued as scheduled in line with National guidance. However, in the second week of March the group was formed and daily meetings were held with core membership as above. The main drivers for this were: (1) major reconfiguration of anaesthetic rotas to provide support and cover of the anticipated surge in Intensive Care Unit (ICU) admissions due to COVID-19, (2) significant reduction in critical care capacity for elective cancer surgery, (3) redeployment of theatre and recover staff usually available for cancer surgery.

Each specialty was asked to submit their five most urgent cases daily. The submitted cases were prioritised by the group and these were mapped to available anaesthetic staff, critical care capacity and surgical theatre space. Cases were then booked for the following day accordingly and work was delivered by NUH surgeons and anaesthetists in a flexible manner. Cases not allocated were brought back as part of the next day’s top five cases from each specialty.

Prior to the relatively late introduction of national pre-screening guidance that changed over the course of the study interval, elective patients were screened for COVID-19 symptoms on the day of surgery by the format of a temperature check and questionnaire relating to the common symptoms of SARS-CoV-2 (new continuous cough, loss in sense of smell or taste and fever During this initial phase, routine pre-operative PCR swab testing was not widely available. Our NHS sites developed separate areas and pathways for patients with suspected or confirmed COVID-19 to minimise risk of nosocomial spread to other inpatients such as separate emergency resus areas, operating theatres and ICUs [8]. The Park BMI Hospital and Spire Nottingham Hospital allowed the development of clean sites for surgery. Clean sites were defined as areas providing care to patients who had been previously screened and tested negative by PCR for COVID-19 infection prior to admission and therefore only accepted patients who were planned for surgery and did not accept acute referrals. The Park BMI Hospital comprised 4 operating theatres, critical care capacity with independent anaesthetic and critical care staff, as well as independent post-operative nursing and allied healthcare professional staff pre-operative assessment. The post-operative sharing of documentation was rapidly established between the Independent Sector and NHS Trust allowing integrated patient care through electronic records.

### Phase 2: 26th March–1st of April

Daily meetings continued and the group moved to weekly allocations. In response to decreased capacity at NUH due to an increasing COVID burden additional anaesthetic, critical care, nursing and junior doctor support was provided to the Independent Sector to start more complex operating. In line with National guidance pre-operative chest radiographs were introduced on the day of surgery [3].

### Phase 3: 1st April–20th of April

Daily COVID Cancer group meetings continued during this phase. Pre-operative imaging was removed with patients being asked to self-isolate for seven days prior to surgery. At the end of this time period 48-h pre-operative COVID-19 swabs were introduced in line with National guidance. These swabs were undertaken in a drive through centre established at the NHS Trust Genitourinary Medicine site and results were confirmed to the patient by text message. Due to the continued burden on NUH critical care services due to COVID-19 at this time, most planned surgery was moved to the Independent Sector. Critical care capacity in the private hospital was further increased with an additional seven perioperative care beds. Approaches from other local Trusts to aid with cancer surgery capacity were received at this time on an individual specialty basis and plans put in place to prioritise these patients through the COVID Surgery group after NUH cancer MDT review.

### Phase 4: 21st April–1st of May

Meetings of the COVID Cancer group reduced to three days a week. Lists were planned up to 2 weeks in advance to comply with 7-day isolation periods and latterly moved towards 14 days in accordance with National guidance. All patients were swabbed 48 h prior to admission for surgery. There was an increase in critical care capacity at the Independent Sector therefore allowing increased capacity for more complex cases to be undertaken. During this time some cases were undertaken in NUH because logistical complexity or risks of surgery precluded use of the private sector, and these factors were felt to outweigh the risk of COVID-19.

### Exposure and outcome definitions

All patients undergoing planned surgery at NUH from 1st of March until 30th of April 2020 were included in a prospectively collected database (Audit Registration 20-190C). Any COVID-19 infection was confirmed by a positive PCR swab from our central laboratory. Suspected cases included those with positive radiological signs confirmed on CT chest or chest radiographs but no confirmatory swab. All-cause 30-day mortality was confirmed from the death date recorded on our electronic record system along with reporting by each specialty to the group. COVID-related mortality also included any mortality following a positive COVID swab where the death was judged to be directly due to COVID within thirty days of COVID diagnosis. All records were searched for further deaths via the NHS spine which is updated every 2 weeks following discharge for patients from out of area. Co-morbidity was defined from hospital discharge records using ICD-10 coding and classified using the Charlson index into 0, 1 or 2 or more co-morbidities [9]. Age at time of surgery and gender were recorded form the electronic health records. Length of stay was determined from electronic hospital admission and discharge records.

### COVID-19 infection

To place the surgical outcomes in context, the UK COVID-19 infection rates are presented using routinely collected data [10]. The numerator being the number of COVID-19 PCR positive cases recorded by local government area and the mid-year populations of each local government area were used to calculate incidence of infection. In addition, all COVID-19 positive patients identified at the Trust by PCR were used to show the local inpatient COVID-19 burden during the time period by day of diagnosis.

### Statistical analysis

Categorical data were summarised using frequencies and percentages. Medians are presented with the interquartile range (IQR). Comparisons were made between categorical data using Chi Squared or Fisher’s exact tests. All statistics were performed using Stata 16 SE (StataCorp. 2019. *Stata Statistical Software: Release 16*. College Station, TX: StataCorp LLC). Tests of significance were considered significant if a *p* value of less than 0.05 was obtained.

## Results

### Overview

**Fig. 1:**
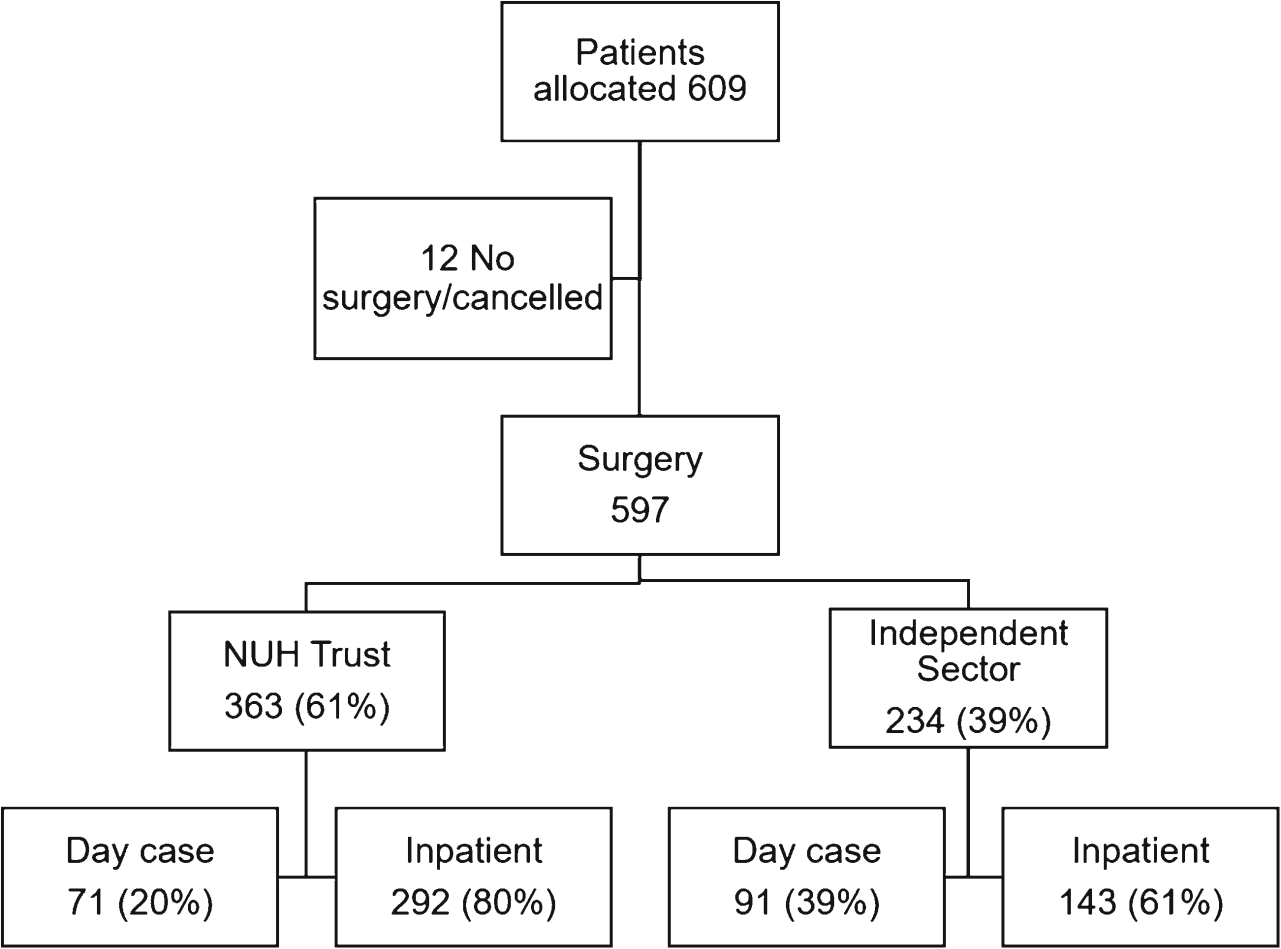
Flow diagram of patients managed during the period

During the study period, 597 patients underwent a surgical procedure. Of these 363 (60.8%) were at NUH and 234 (39.2%) in the Independent Sector (Fig. 1) with no fatalities of oncology and urgent surgical patients on the waiting list. In total, 162 (27.1%) were day case procedures. The split of cases by specialty is shown in Table 1. The median age of patients was 65 years (IQR 54–74 years). In total, 51.8% of operations were undertaken in males. A cancer diagnosis was present in 85.9% (513/597) of cases with 87.9% (525/597) having a Charlson score of one or more. During the time period, 92 patients had a COVID-19 swab prior to admission and none were positive.

**Table 1:** Operative procedures by specialty across sites

Specialty	NUH (*n* (%))	Independent Sector (*n* (%))
Breast	19 (23.5)	62 (76.5)
Colorectal	34 (81.0)	8 (19.0)
Head and neck	44 (77.2)	13 (22.8)
Endocrine	4 (28.6)	10 (71.4)
Gynaecology	35 (87.5)	5 (12.5)
Hepatobiliary	12 (46.2)	14 (53.8)
Neurosurgery	13 (100)	0 (0)
Other	29 (76.3)	9 (23.7)
Plastics	35 (53.0)	31 (47.0)
Urology	72 (50.7)	70 (49.3)
Upper GI	12 (63.2)	7 (36.8)
Thoracics	54 (91.5)	5 (8.5)

Patients operated on in the Independent Sector were younger with a median age of 62 years (IQR 52–73 years) compared to 67 years (IQR 55–74 years) in NUH. The patients in the Independent Sector were also less likely to have co-morbidity and were more likely to be females. A greater proportion of thoracic-abdominal procedures were undertaken at NUH than in the Independent Sector (Table 2). Length of stay following thoraco-abdominal resection was 5 days (IQR 3–7 days).

**Table 2:** Comparison of cases performed at the Independent Sector and NUH

	NUH	Independent Sector	*p* value
Age	67 years (IQR 55–74)	62 years (IQR 52–73)	<0.05
Sex F/M	157/206	131/103	<0.05
Co-morbidity >1	89.8% (326/363)	85.0% (199/234)	<0.05
Cancer %	89.3% (324/363)	80.8% (189/234)	<0.05
Abdomino/thoracic	34.4% (125/363)	19.2% (45/234)	<0.05

### Surgical outcomes

#### COVID-19 diagnoses

##### PCR only positive

During the time period, 1.3% (8/597) of patients tested positive for COVID-19 following surgery of which 50% were male (4/8), 87.5% (7/8) had a cancer diagnosis or co-morbidity score >1 and all had undergone surgery at NUH. Five of these patients were diagnosed during their initial inpatient stay and three tested positive following discharge. Patients who tested positive for COVID-19 had a median age of 69.5 years (IQR 58–74.5 years) and length of stay 13.5 days (IQR 2.5–16 days). Median time from surgery to diagnosis of COVID-19 infection by PCR test was 6 days (IQR 4–22.5 days) with five patients presenting symptomatically. Three patients who tested positive by PCR for COVID-19 infection did not receive a pre-operative PCR swab and therefore whether these patients were asymptomatic prior to surgery or developed nosocomial infection is unclear. In patients having major resections, all of which were abdominal/thoracic, 3.5% (6/170) developed COVID-19 post-operatively. All these patients were managed on the surgical ward with no escalation to critical care.

##### COVID-19 suspected on imaging

Following surgery 97 had a post-operative CT scan and 86 had a post-operative chest radiograph. This showed that in addition to the PCR positive patients, a further ten patients had changes suspected to be COVID-19 on post-operative imaging. Seven of these cases were at NUH and three at the Independent Sector site with none requiring admission to critical care. Six were post-operatively PCR tested all with a negative COVID-19 result; the four remaining patients were not PCR tested as this investigation was not available during their perioperative period.

##### Overall COVID-19 diagnoses

In total, combining those cases positive on PCR and those with suspected COVID-19 on post-operative imaging 3.0% (18/597) of patients operated on during the period developed confirmed or suspected COVID-19. In the Independent Sector, 1.3% had a positive swab or suspected diagnosis compared to 4.1% in the Trust (*p*=0.047).

#### COVID-19 related mortality

Thirty-day all-cause mortality overall was 0.7% (4/597) with one (0.2%) patient dying within 30 days following a COVID-19 positive diagnosis. The COVID-19 related 30-day mortality was therefore 12.5% (1/8) of PCR COVID-19 positive patients and 5.6% (1/18) of radiologically suspected or PCR confirmed diagnosis of COVID-19. This single death, believed in part attributable to surgery in combination with COVID-19 infection, occurred following discharge at another centre having had a positive post-operative swab for COVID at NUH.

A second patient who had a positive post-operative COVID-19 swab died beyond 30 days but shortly after testing positive for COVID-19. However, this patient was considered palliative at the time and so although COVID-19 infection may have contributed to the death, the impact of surgery on contribution to mortality is debated. The overall mortality to date beyond 30 days was therefore 25.0% (2/8) for a PCR positive COVID-19 diagnosis, and 11.1% (2/18) for radiologically suspected or PCR confirmed diagnoses of COVID-19.

### Local infection rates

**Fig. 2:**
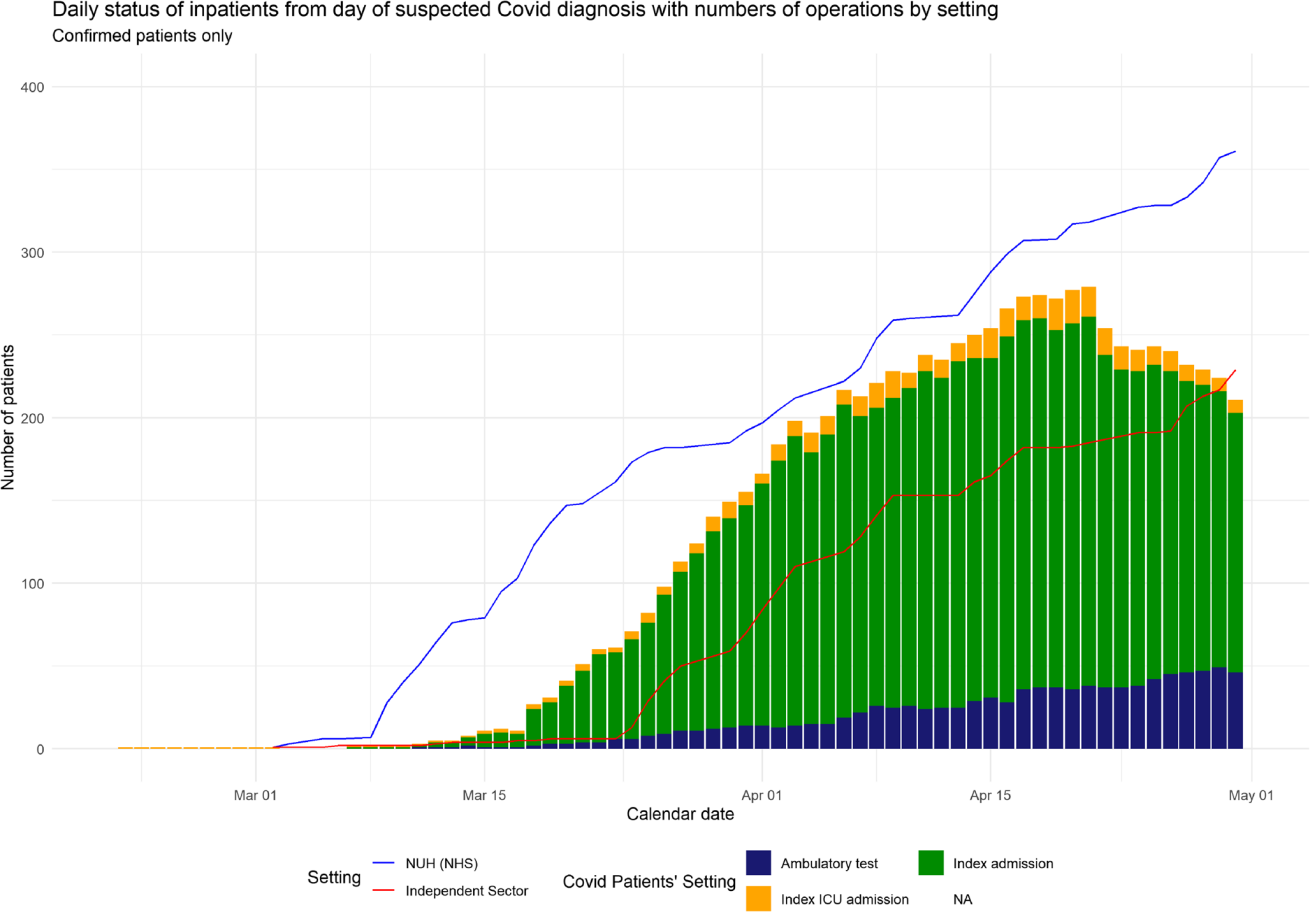
Daily status of inpatients from day of suspected COVID diagnosis with numbers of operations by setting

Figure 2 shows the cumulative number of operative cases performed per site with the number of cases of PCR positive COVID-19 infection hospitalised at NUH during the time period by day. There were no cases of COVID-19 managed at the clean Independent Sector sites.

## Discussion

**Fig. 3:**
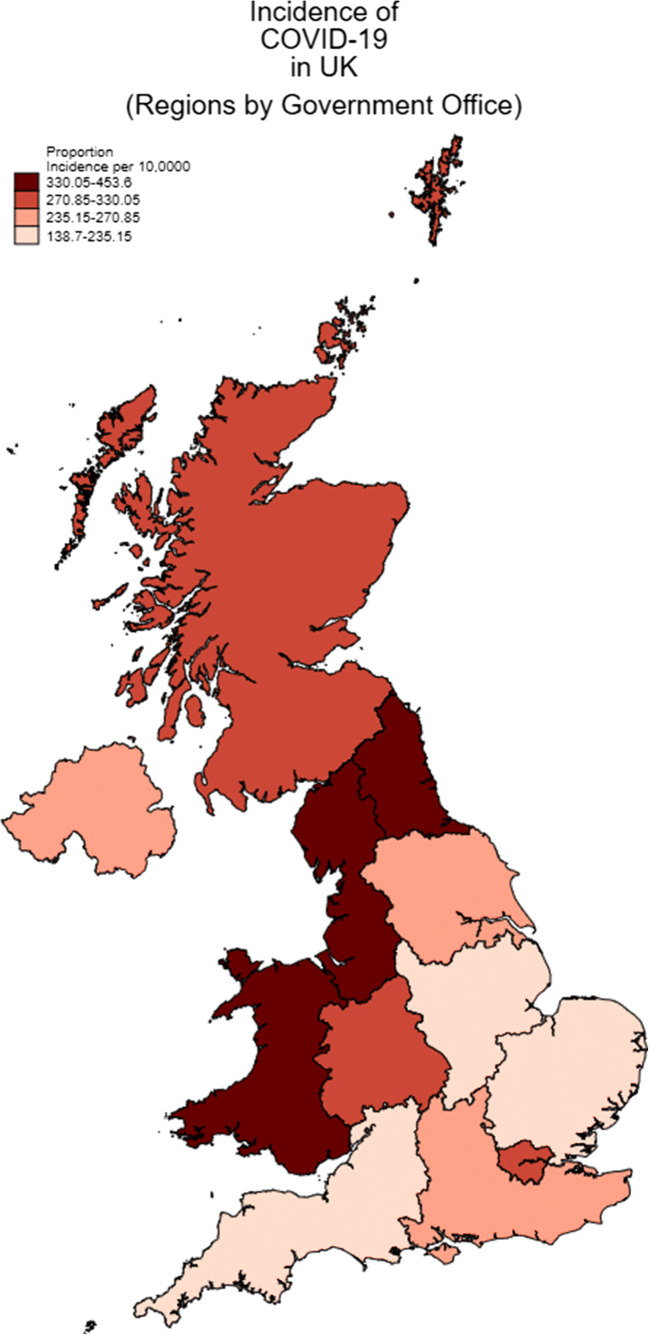
Map of UK incidence of COVID by local Government region by 4th June 2020

Following planned urgent and cancer surgery we report an overall and major resection post-operative COVID-19 infection rate of 1.3% and 3.5% with no PCR confirmed cases at our clean site. Overall mortality was low and only two deaths occurred within 30 days following confirmed or suspected COVID-19 diagnosis. During the study time period, the incidence of COVID-19 in the East Midlands was 193.7 per 100,000 population and the second lowest by local government area in the UK (Fig. 3) [10]. In an area of low incidence of COVID-19 infection, we have demonstrated that planned urgent and cancer surgery can be continued in appropriately counselled patients. This relies on robust systems to prioritise cases and appropriate resources to undertake surgery. The use of a clean site does offer additional safety than that achieved despite precautions in our Trust estate, reflecting the challenges of segregating patients in an acute Trust with throughput of non-elective non-isolated inpatients. Access to the Independent Sector enabled us to establish safe pathways, with increased transfer of services as the pandemic peaked to a clean site with no post-operative cases of confirmed COVID infection.

Patients were identified prospectively and their results audited in real time to ensure that adverse outcomes were avoided. It is possible that patients following surgery had asymptomatic COVID infections so the true rate of infection may be greater than we report, however all patients with suspected diagnoses were swabbed or imaged during the study period. It is possible that patients presented to sites outside of the Trust and obtained a diagnosis post discharge. This study is further limited by its single centre design and potential extrapolation of results due to the low overall numbers COVID-19 diagnoses. Therefore, larger multicentred prospective cohorts could provide more robust data although the feasibility of ensuring multiple institutions conform to the same prioritisation group standard would pose significant challenges.

Our mortality following a diagnosis of COVID-19 in the post-operative period is 25% in line with reported studies; however, this is set against a low infection rate following surgery [2]. In turn, our low infection rate following surgery should be contextualised by a low incidence in the surrounding area. However, even with this low incidence, local critical care capacity was exceeded at the peak of the infection curve requiring more than a doubling of ventilated beds across the Trust. This would have prevented all operating had the Independent Sector not been mobilised given the redeployment of theatre and anaesthetic staff to cover these additional beds. The formation of the COVID cancer group enabled cases to be prioritised based on clinical and ethical grounds and utilise the resources available in the most equitable way. A similar model of care has been reported in other areas [11].

The current study occurred during the most significant clinical event in the NHS since its inception. It was a constantly and rapidly evolving situation with new evidence and guidance becoming available on an almost daily basis [3]. Some of this was based on experience in other health care systems, particularly China and Italy, where the pandemic had an earlier timeline [12–14]. This resulted in a constantly changing approach from the surgical and perioperative teams. The situation continues to evolve and the results presented here represent only a snapshot of the risks and safety of surgery within the pandemic environment. Whether this reassurance remains applicable for future practice is uncertain.

These data should be particularly relevant in the context of the NHS’s restoration plans [15]. All the cases described herein involved the treatment of patients with either cancer or other urgent pathology. The risk of disease progression or even risk to life in the context of approximately 1% infection risk is likely to be acceptable to most patients and clinicians. This becomes less acceptable when the indication for surgery is focused on symptom control or other quality of life indications, and the overall balance between benefit and risk narrows. Additional surgical throughput also adds greater strain on prevention measures such as PPE usage, capacity to test and social distancing. The last of these also has a potentially significant impact on the bed availability in NHS Trusts overall. Adherence to pre-operative self-isolation for 7 days and latterly 14 days has been a key pillar of our pathway but as the government lifts lockdown, patients and their families might find this difficult to maintain, especially in those of working age. Parents and carers of the paediatric surgical population who have hitherto been unable to work may find this particularly challenging.

Despite the relatively small sample size the risk of infection between the clean private sector site and our NUH site appears substantially different. There are many confounding factors such as volume, age, co-morbidity, complexity of surgery and overall length of stay. However, the lack of confirmed infection within the dedicated clean site, without any non-elective admissions, remains stark. Our environment within the Independent Sector during this study would be described as “E1” by the Association of Coloproctology of Great Britain and Ireland [16]. By contrast our Trust would be considered an “E3” environment during this study period. Which components of a “clean” environment are of significance is unclear. It is likely that this might be multi-factorial, with each component in combination providing more than the sum of its part. This study however is currently the only evidence of the advantages a true “E1” environment in a UK healthcare setting and should help direct future plans for restoration of surgical services. NHS Trusts with estates that allow sites without any non-elective admissions may be able to reproduce similar outcomes to our Independent Sector site if local COVID-19 prevalence falls, but where this is not possible continued collaboration across all healthcare provides might be necessary. Equally falling COVID-19 prevalence may enable similar outcomes in the acute NHS and may be transferable to regions outside the NHS globally under similar comparable conditions but this is by no means guaranteed.

## Conclusion

The use of a prioritisation group along with provision of clean site operating has enabled us to continue to operate on our urgent and cancer cases during the current pandemic. The rates of COVID-19 infection following surgery were low but the mortality associated with this infection was high and patients need to be consented appropriately for this significant novel post-operative complication.

## Supplementary Information

ESM 1 (DOCX 26 kb)

ESM 2 (DOC 83 kb)

ESM 3 (TIFF 2949 kb)

High Resolution Image (PNG 38392 kb)

## Data Availability

Anonymised data available on request, raw data not available due to patient confidentiality.

## References

[1] Nepogodiev D, Bhangu A (2020) Elective surgery cancellations due to the COVID-19 pandemic: global predictive modelling to inform surgical recovery plans. Br J Surg 10.1002/bjs.11746 PMC7272903 32395848

[2] Mortality and pulmonary complications in patients undergoing surgery with perioperative SARS-CoV-2 infection: an international cohort study. Lancet (London, England)*.* 2020 10.1016/S0140-6736(20)31182-X PMC7259900 32479829

[3] COVID-19 guidance for surgeons working during the pandemic. https://www.rcsengacuk/coronavirus/joint-guidance-for-surgeons-v2/. Accessed June 2020

[4] Intercollegiate guidance for pre-operative chest CT imaging for elective cancer surgery during the COVID-19 pandemic. Royal College of Surgeons of England https://www.rcsengacuk/coronavirus/preoperative-chest-ct-imaging-guidance/. Accessed June 2020

[5] Moletta L, Pierobon ES, Capovilla G et al International guidelines and recommendations for surgery during Covid-19 pandemic: a systematic review. International journal of surgery (London, England). 2020 10.1016/j.ijsu.2020.05.061 PMC7245259 32454253

[6] Iacobucci G (2020). Covid-19: all non-urgent elective surgery is suspended for at least three months in England. BMJ (Clinical research ed).

[7] Turaga KK, Girotra S Are we harming cancer patients by delaying their cancer surgery during the COVID-19 pandemic? Annals of surgery*.* 2020 10.1097/SLA.0000000000003967 PMC7299109 32487802

[8] Flemming S, Hankir M, Ernestus RI (2020). Surgery in times of COVID-19-recommendations for hospital and patient management. Langenbeck's Arch Surg.

[9] Charlson ME, Pompei P, Ales KL, MacKenzie CR (1987). A new method of classifying prognostic comorbidity in longitudinal studies: development and validation. J Chronic Dis.

[10] Number of coronavirus (COVID-19) cases in the United Kingdom. https://www.statistacom/statistics/1102151/coronavirus-cases-by-region-in-the-uk/. June 2020

[11] Qadan M, Hong TS, Tanabe KK, Ryan DP, Lillemoe KD (2020) A multidisciplinary team approach for triage of elective cancer surgery at the Massachusetts General Hospital during the novel coronavirus COVID-19 outbreak. Ann Surg 10.1097/SLA.0000000000003963 PMC7188033 32301804

[12] Cai M, Wang G, Zhang L (2020). Performing abdominal surgery during the COVID-19 epidemic in Wuhan, China: a single-centred, retrospective, observational study. Br J Surg.

[13] Torzilli G, Vigano L, Galvanin J et al (2020) A snapshot of elective oncological surgery in Italy during COVID-19 emergency: pearls, pitfalls, and perspectives. Ann Surg 10.1097/SLA.0000000000004081 PMC7373476 32675512

[14] Low TY, So JBY, Madhavan KK, Hartman M (2020) Restructuring the surgical service during the COVID-19 pandemic: experience from a tertiary institution in Singapore. Br J Surg 10.1002/bjs.11701 PMC7272885 32406932

[15] Fields AC, Vacanti JC, Rhee C et al (2020) Restarting essential surgery in the era of COVID-19: a cautious data driven approach based on the literature and local data. Ann Surg 10.1097/SLA.0000000000004109 PMC7467051 32452948

[16] Resumption of elective colorectal surgery during COVID-19 ACPGBI *The Association of Coloproctology of Great Britain and Ireland* https://www.acpgbiorguk/content/uploads/2020/04/ACPGBI-considerations-on-resumption-of-Elective-Colorectal-Surgery-during-COVID-19-v28-4-20pdf. Accessed 2020 10.1111/codi.15282 32726872

