# Supplementary material for: Planned surgery in the COVID-19 pandemic: a prospective cohort study from Nottingham
Source: Langenbecks Arch Surg. 2021 Jun 15;406(7):2469–77. doi: 10.1007/s00423-021-02207-8 (PMC8204733; doi:10.1007/s00423-021-02207-8)
Supplement: Supplementary file 1 — (DOCX 26 kb) [file 423_2021_2207_MOESM1_ESM.docx]

Nottingham University Hospitals NHS Trust

TERMS OF REFERENCE – COVID CANCER PRIORITISATION MDT

| Purpose | To ensure NUH has a dedicated senior clinical forum to prioritise limited NUH theatre and critical care capacity for cancer patients during the SARS-CoV-2 pandemic. |
| --- | --- |
| Chair | Cancer Lead Clinician  Deputy |
| Attendees | Deputy Medical Director  Operations – Deputy COO  Head of Cancer Performance  Cancer Lead Nurse  Clinical Support (Anaesthetics and Critical Care)  Sixth division – pre-op  Surgery  Family Health  CAS  Ethics – lay representatives of NUH’s Ethics of Clinical Practice Committee (who will ask questions and offer ethical principles but who will not take part in any individual patient decisions) |
| Restrictions | The meeting shall not commence unless it is quorate. No quorate meeting shall continue if it subsequently becomes non-quorate. |
| Quorum | 2 Surgeons  A Representative from Anaesthetics  A representative from Critical Care |
| Secretary | Minutes recorded |
| Frequency and Venue | Daily (Monday – Friday and Sunday) 1-2pm  Silver Command Suite – QMC and City with dial in facility (at weekend) and remote VTC when available  Remote dial number Silver Command |
| Papers | 1. List of Divisions’ priority patients to be submitted by Theatres to group by 11am daily (based on Divisional submission by 10am). 2. Availability of theatres and critical care to be submitted by Theatres by 11:30am daily. 3. Decisions to be made with reference to simplified series of questions below to aid prioritisation that came out of the MORAL Balance analysis work. 4. Decisions can extend to urgent patients where necessary in addition to cancer patients where required however paediatrics/neuro-oncology/trauma/Spine patients and specialty emergency patients are excluded from this decision-making meeting. 5. Detailed list of patients prioritised will be sent out after the meeting and notes of meeting circulated the same day |
| Reporting Arrangements | COVID Incident Command Structure |
| Duties | - To review priority list and decide which patients will be prioritised for the following day’s capacity. The priority list must be complete providing evidence patients have been worked up and ready to proceed to surgery with clear clinical rationale which must be available on priority list from divisions. - To decide rules to guide prioritisation of cases on a longer time scale - To consider ethical implications of these decisions. - To communicate these decisions to Divisions promptly for action. - To ensure that explanation is provided to waiting patients and their families. - To consider any other requests related to cancer activity e.g. 2ww prioritisation. - To ratify daily allocation of any private sector capacity (decision made outside of meeting and coordinated by Chair) and ensure no duplication. |
| Approval and Review | Approved: 26 March 2020  To be reviewed: 30 June 2020 |

**Question list-/guide for members**

1. Can the surgery wait? Are other treatment options possible? If yes, do not list.
2. Proposed benefit of the surgery - cure (likelihood) vs palliation? If palliation do not list.
3. Complexity of the surgery:
   1. Pre-op treatment needed e.g. chemotherapy, radiotherapy?
   2. Duration of surgery?
   3. Duration of expected hospital stay?
   4. Special resources needed intraoperatively (people or equipment)?
   5. Postoperative need - critical care level 2 or 3? Specialised ward?
   6. Complication risk – especially need for mechanical ventilation?
   7. Is there sufficient care on hospital discharge in the community?
   8. Is this aerosol generating surgery?
4. Individualised patient factors:
   1. Estimated consequence to the patient of a surgery delay?
   2. Likelihood of patient making a swift recovery, without complications (co-morbidities, frailty and physiological reserve)?
   3. Any other individual patient factors that have relevance?

**Group needs to consider for prioritisation**

1. Local COVID-19 risk in hospital and community?
2. What theatre capacity has been allocated for cancer surgery (public and private), on each campus?
3. What ICU / HDU capacity is available?
4. Efficiency considerations (Goal is to maximise efficiency by maximum throughput for maximum patient benefit):
   1. Complexity of surgery intraoperative?
   2. Complexity of surgery postoperative?
   3. Delays from swapping surgical specialties during a theatre session.

Once priority decided there needs to be consideration of how this is explained to the patient especially those still waiting.
