# Supplementary figures and images for: Planned surgery in the COVID-19 pandemic: a prospective cohort study from Nottingham
Source: Langenbecks Arch Surg. 2021 Jun 15;406(7):2469–77. doi: 10.1007/s00423-021-02207-8 (PMC8204733; doi:10.1007/s00423-021-02207-8)

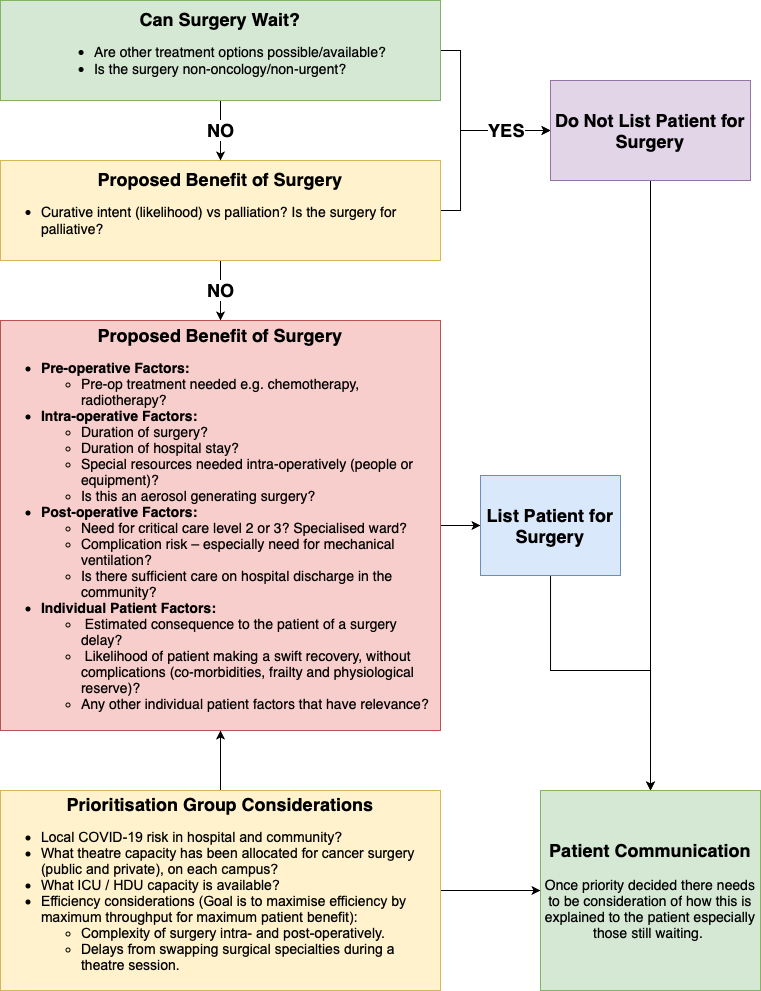

Supplement: Supplementary file 3 — (TIFF 2949 kb) [file 423_2021_2207_MOESM3_ESM.tiff]

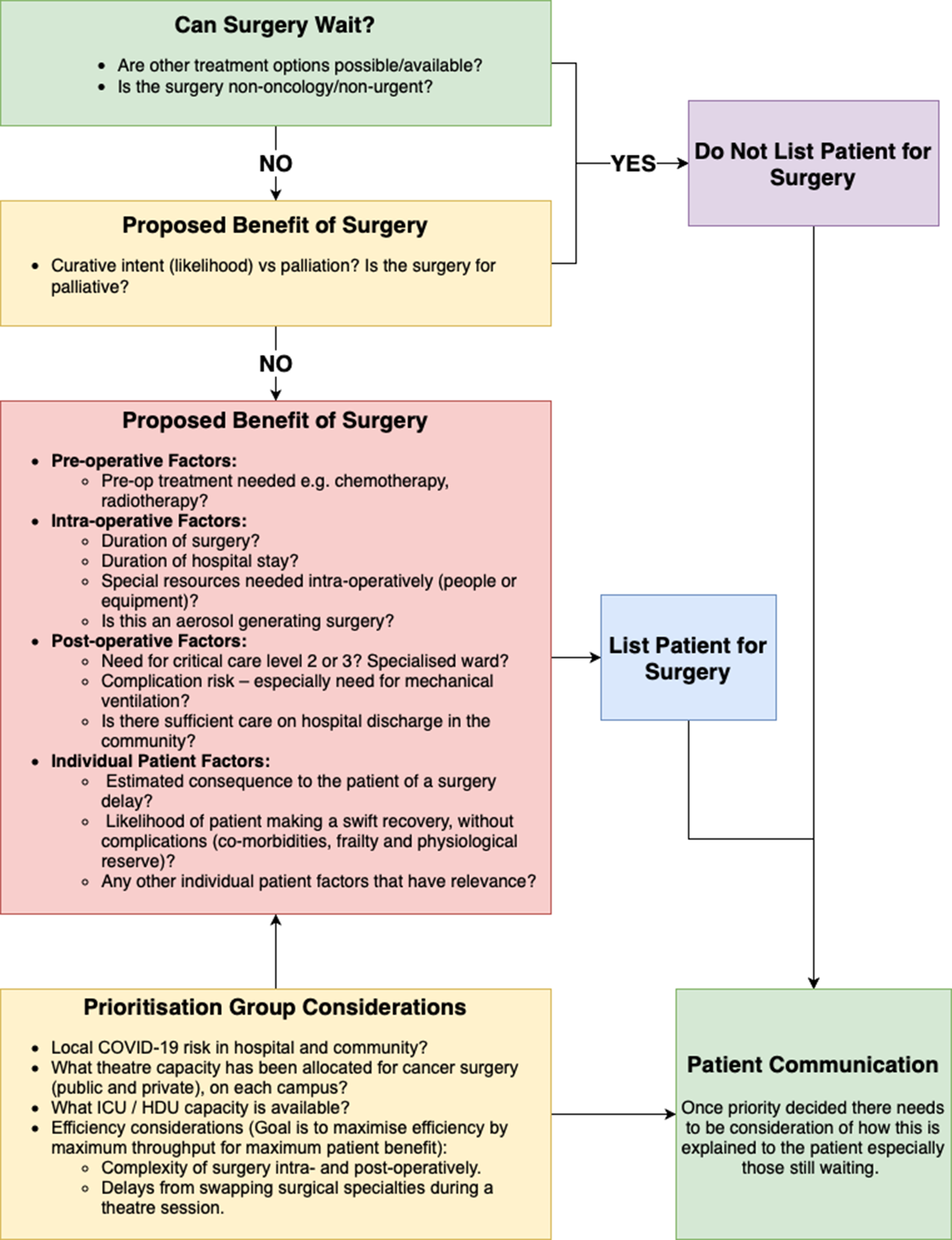

Supplement: Supplementary file 4 — High Resolution Image (PNG 38392 kb) [file 423_2021_2207_FIG4_ESM.png]
